# Organic affective disorder due to meningioma, case report

**DOI:** 10.1192/j.eurpsy.2023.837

**Published:** 2023-07-19

**Authors:** M. Garcia-Moreno, A. de Cós Milas, L. Beatobe Carreño, P. del Sol Calderón, Á. Izquierdo de la Puente

**Affiliations:** 1 HOSPITAL UNIVERSITARIO PUERTA DE HIERRO MAJADAHONDA; 2 HOSPITAL UNIVERSITARIO DE MÓSTOLES, MADRID, Spain

## Abstract

**Introduction:**

Brain tumors can be associated with psychiatric symptoms in up to 50% of cases. The most frequent primary is meningioma and the clinic will depend on its location. Since surgical treatment does not always guarantee complete resolution of the condition, concomitant psychopharmacological treatment is usually recommended.

**Objectives:**

To review about organic mania and its differential diagnosis.

**Methods:**

We carry out a literature review about organic affective disorder accompanied by a clinical description of one patient with organic mania.

**Results:**

A 50-year-old woman admitted due to psychotic symptoms. She had a diagnosis of frontal and parietal meningioma treated with surgical treatment 10 years ago. In this context she had a diagnosis of Organic Affective Disorder and 3 previous psychiatric admissions due to affective or psychotic symptoms. Current episode consisted in dysphoria, magalomanic ideation, delusional ideation of harm and mystical-religious content, high speech pressure and insomnia with little awareness of the disease. Cranial magnetic resonance showed postoperative right frontal changes and stability in parietal meningioma, with no significant differences compared to the previous study. Diagnosis of Organic Affective Disorder is maintained and reintroduced treatment with aripiprazole withdrawaled by the patient weeks before. Because of adverse effects and persistence of the symptoms described, it was changed to olanzapine with good response and tolerability. The behavior was progressively adapted with improvement of the dysphoria and without psychotic symptoms at discharge.

**Conclusions:**

Affective symptons due to organic disorders such as brain tumors can be treated surgically and with psychopharmacological treatment.

**Disclosure of Interest:**

None Declared

